# Simultaneous evaluation of intermittency effects, replica symmetry breaking and modes dynamics correlations in a Nd:YAG random laser

**DOI:** 10.1038/s41598-022-05090-5

**Published:** 2022-01-20

**Authors:** Edwin D. Coronel, Manoel L. da Silva-Neto, André L. Moura, Iván R. R. González, Roberta S. Pugina, Eloísa G. Hilário, Euzane G. da Rocha, José Maurício A. Caiut, Anderson S. L. Gomes, Ernesto P. Raposo

**Affiliations:** 1 Departamento de Física, Universidade Federal de Pernambuco, Recife, PE 50670-901 Brazil; 2 Graduate Program in Materials Science, Universidade Federal de Pernambuco, Recife, PE 50670-901 Brazil; 3 Grupo de Física da Matéria Condensada, Núcleo de Ciências Exatas–NCEx, Universidade Federal de Alagoas, Campus Arapiraca, Arapiraca, AL 57309-005 Brazil; 4 Laboratório de Física Teórica e Computacional, Departamento de Física, Universidade Federal de Pernambuco, Recife, PE 50670-901 Brazil; 5 Unidade Acadêmica de Belo Jardim, Universidade Federal Rural de Pernambuco, Belo Jardim, PE 55156-580 Brazil; 6 Departamento de Química, Faculdade de Filosofia, Ciências e Letras de Ribeirão Preto, Universidade de São Paulo, Ribeirão Prêto, SP 14040-901 Brazil

**Keywords:** Physics, Optical physics

## Abstract

Random lasers (RLs) are remarkable experimental platforms to advance the understanding of complex systems phenomena, such as the replica-symmetry-breaking (RSB) spin glass phase, dynamics modes correlations, and turbulence. Here we study these three phenomena jointly in a Nd:YAG based RL synthesized for the first time using a spray pyrolysis method. We propose a couple of modified Pearson correlation coefficients that are simultaneously sensitive to the emergence and fading out of photonic intermittency turbulent-like effects, dynamics evolution of modes correlations, and onset of RSB behavior. Our results show how intertwined these phenomena are in RLs, and suggest that they might share some common underlying mechanisms, possibly approached in future theoretical models under a unified treatment.

## Introduction

Random Lasers (RLs) have become valuable photonics sources for a diversity of basic and applied studies since their conception by Letokhov in 1966^1^, followed by experimental studies of stimulated emission with Nd^3+^ ions^2,3^, and the definitive demonstration of laser action in a scattering medium in 1994^4^. RLs differ from regular lasers in the optical feedback mechanism. Whereas in regular lasers the feedback is provided by a static cavity generally formed by two mirrors, in RLs the optical feedback comes from the stimulated photons in the gain medium that are randomly scattered in a disordered material.

Since 1994, several possibilities of different pump sources have been demonstrated^5–11^, including optical and electrical pumping associated with gain media, such as liquid dyes, semiconductors, quantum dots, polymers, biotissues, and rare-earth doped crystals and glasses. The scattering media can be passive, like TiO_2_ or Al_2_O_3_, metallic, as gold or silver nanostructures, which allow plasmonic enhancement, or active materials as semiconductors and rare-earth doped ions.

Here we are interested in Nd^3+^ based RLs, which are among the pioneering materials employed in the quest for RL emission^2,3,12–14^. Being an active material, the Nd^3+^ ions randomly incorporated in micron- or nano-powders based on glass or crystal hosts^15–21^ act simultaneously as gain and scattering media. Although the Nd^3+^ ion can be excited in the visible to NIR (600–900 nm) range^17,18^, with the most important emission near 1064 nm, excitation around 808 nm is mostly employed due to the availability of powerful semiconductor laser sources, besides being one of the strongest absorption transitions in Nd^3+^, though with a relatively large quantum defect^17,18^. Nd-based RLs have been exploited along with second order optical nonlinearities in the crystal nanopowder host to demonstrate tunability through self second harmonic and sum-frequency generation^19^. They have already found important applications as speckle free sources for imaging^22^ and nanothermometers^23^.

Another relevant application of RL systems in general, and Nd-based RLs in particular, concerns the multidisciplinary field of complex systems. In ref.^24^ Gomes and co-workers demonstrated the simultaneous observation of Lévy-like statistics of emitted intensities and the replica symmetry breaking (RSB) phenomenon in Nd-based RLs. Lévy statistics is typical of systems exhibiting strong fluctuations, which are generally not accounted for by considering conventional statistical physics models based on weak Brownian fluctuations dynamics and the central limit theorem^24^. In photonics, the first observations^25,26^ of Lévy statistics in RLs were followed by a number of theoretical and experimental studies on several RL systems, including distinct gain media and spatial dimensionalities^24–32^.

RSB is a phenomenon which was first experimentally demonstrated in RLs by Ghofraniha and co-workers^33^, and since then has attracted a lot of attention^34–36^, with reports in different RL schemes and gain-scatterers combinations. The concept of RSB, introduced by Parisi in the context of disordered magnetic spin glasses^37,38^, refers to the property that identically-prepared systems (i.e., replicas) can reach distinct states and lead to distinct measurements of observables. In a series of seminal works (see the review^38^ and references therein), a photonic glassy phase with RSB was predicted in the RL regime above threshold, in which the modes cannot oscillate in a synchronous way, presenting nontrivial correlations captured by the Parisi overlap parameter.

Although RLs are cavity-less, they are not modeless, and this feature has instigated a great deal of theoretical and experimental research, showing that a RL is in fact a multimode laser^39–47^. One of the statistical tools employed to analyze modes correlations in photonic systems is the Pearson correlation coefficient. Regarding RLs, the Pearson coefficient was first applied^44^ to study the emission of a nanofiber RL system with and without TiO_2_ nanoparticle scatterers, whereas in^45^ the mode-locking transition was investigated by varying the excitation beam profile in a RL with TiO_2_ nanoparticles in Rhodamine dye solution. More recently, Sciuti and co-workers^46^ studied the modes dynamics in the mode-locking transition via the temporal mapping of the emitted spectra characterized by the Pearson correlation in a RL based on dye-doped electrospun nanofibers. Also, Sarkar and co-workers related^47^ the modes correlations to the RSB and Lévy-like properties of a weakly scattering optofluidic RL. Additionally, our group have also recently applied the Pearson analysis to a hybrid electronically addressable random fiber laser^48^.

Another striking complex feature recently demonstrated^49^ in a random fiber laser is the turbulent-like behavior of RL emission. In this context, we have shown^50^ that a modified Pearson measure can account simultaneously for the coexistence of turbulence-like and RSB glassy behaviors in a random fiber laser.

In this work, we demonstrate that the joint analysis of the modes dynamics correlations, RSB phenomenon, and intermittency turbulent-like effects can be performed from a couple of modified Pearson coefficients. To apply the theoretical concepts, our experimental study system is a Nd-based RL consisting of Nd^3+^ ions in YAG micron crystal powder (Nd:YAG), synthesized for the first time using a spray pyrolysis method, which enabled us to control the composition of particles and crystalline phase. That fast methodology produces particles in a few minutes, without coalescence and might allow also to produce these materials on a large scale, as required by industry.

From a proper statistical analysis of the intensity fluctuations in the long time series of spectra emitted by the Nd:YAG RL, we characterize, using the proposed measures, either the evolution of the modes correlations, the shift from the photonic paramagnetic-like prelasing regime to the RSB glassy RL phase, and the emergence and fading out of intermittency effects. Remarkably, the simultaneous coexistence of turbulence and RSB in any physical system brings together two of the most intricate current problems in physics, whose theoretical understanding still lacks^51^.

Our results reveal the concurrent onset of enhanced mode correlations, photonic RSB and turbulence behavior in the Nd:YAG RL. We argue that these complex photonic phenomena result from a unique combination of key ingredients, i.e., a suitable disorder strength of gain and scattering elements, spatial overlap of multiple coupled modes, and nonlinearity degree, all of them unveiled in this work from a single set of measurements of intensity fluctuations.

### Nd:YAG micron size powder RL

We employed a spray pyrolysis method to synthesize the particles of Nd:YAG (4.0% mol/mol to Y^3+^), with average diameter of 0.6 μm, see “Methods” section for details.

A series of 5000 spectra for each excitation energy was acquired to study the intermittency effects, and for the analysis of RSB behavior and modes correlations 1000 spectra were obtained in the regimes below, close to, and well above threshold. The pump source was an optical parametric oscillator pumped by the second harmonic of a Q-switched Nd:YAG laser (see “Methods” section).

**Figure 1:**
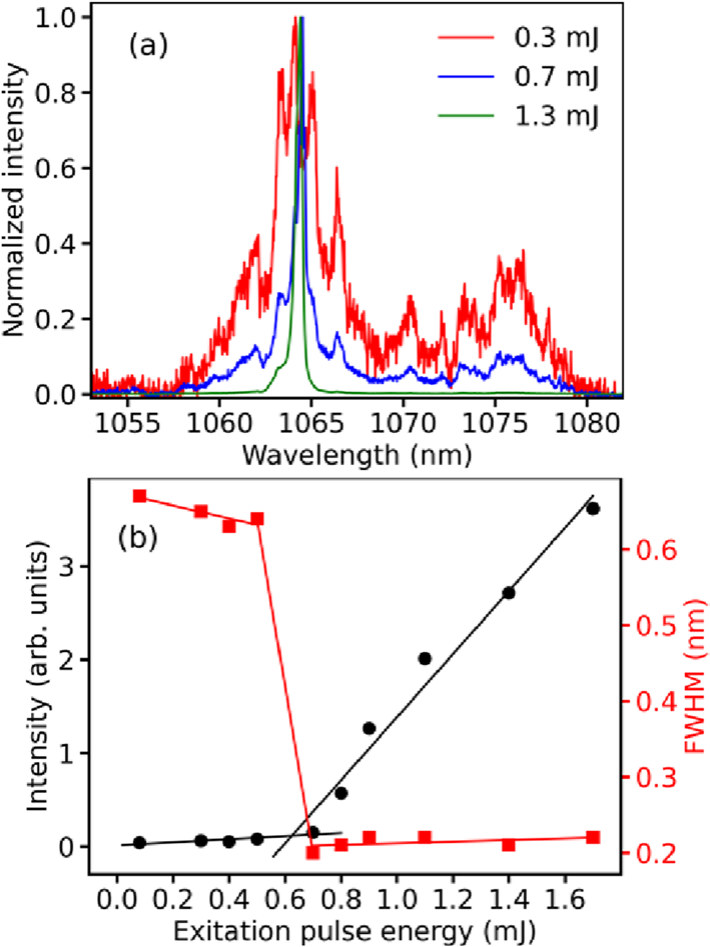
Characterization of the emitted intensity and FWHM of the Nd:YAG RL. (**a**) RL spectral evolution from an excitation pulse energy below the threshold (0.3 mJ) to well above threshold (1.3 mJ). (**b**) Emitted intensity (left y-axis) and FWHM (right y-axis) versus excitation energy. The energy threshold 0.62 mJ is inferred from (**b**).

Figure 1a shows the spectral evolution from an excitation pulse energy below the RL threshold (0.3 mJ) to well above threshold (1.3 mJ) (the energy threshold is 0.62 mJ), whereas Fig. 1b displays the emitted intensity (left y-axis) and FWHM (right y-axis) versus excitation energy. We notice that the emitted spectrum below threshold presents large bandwidth, with the highest intensity maximum occurring at $\lambda = 1064$ nm (main emission of Nd^3+^). As the RL threshold is approached, abrupt narrowing and increasing of the slope efficiency are observed.

## Results and discussion

We start by defining our notation. In order to avoid confusion, we use Greek letters to represent the spectrum labels and small Latin letters to indicate the wavelength labels. We thus denote, for example, $I_{\gamma i}$ as the intensity emitted by the Nd:YAG RL at the wavelength $\lambda_{i}$ in the spectrum $\gamma$, with $\gamma$ integer. Moreover, $I_{i}$ is the intensity at the wavelength $\lambda_{i}$ averaged over the spectra. We also define $\overline{{\Delta_{\gamma i} }}$ as the relative difference (fluctuation) with respect to this average, $\overline{{\Delta_{\gamma i} }} = \Delta_{\gamma i} /\sqrt {\sum\nolimits_{K} {\left( {\Delta_{\gamma i} } \right)^{2} } }$, where $\Delta_{\gamma i} = I_{\gamma i} - I_{i}$ and capital Latin letters represent either the spectra (e.g., $K = \gamma$) or wavelengths (e.g., $K = i$). We then propose a modified Pearson correlation coefficient as follows,

1 $$ P_{MN} = \overline{{ \Delta_{MK} }} \overline{{\Delta_{NK} }} , $$

where above we use the Einstein summation convention over repeated indexes.

The advantage to use this modified Pearson coefficient is that it comprises both the Parisi overlap parameter $q_{\gamma \beta } ,$ which characterizes the photonic glassy RL phase with RSB, and the Pearson correlation $C_{ij}$ between RL modes. Indeed, on the one hand, by setting in Eq. (1) the spectrum indexes $M = \gamma$ and $N = \beta$ and the wavelength index $K = i$, we obtain the Parisi overlap parameter^33^, given by

2 $$ P_{M = \gamma ,N = \beta } = q_{\gamma \beta } = \frac{{\sum\nolimits_{i} {\Delta_{\gamma i} \Delta_{\beta i} } }}{{\sqrt {\left[ {\sum\nolimits_{i} {\left( {\Delta_{\gamma i} } \right)^{2} } } \right]\left[ {\sum\nolimits_{i} {\left( {\Delta_{\beta i} } \right)^{2} } } \right]} }} . $$

In the photonic context, each spectrum is considered a replica emitted by the system, i.e., a copy of the RL signature generated under identical experimental conditions. The probability density function (PDF) $P\left( q \right)$, analogue to the Parisi order parameter in spin glass theory^37,38^, describes the distribution of replica overlap values $q = q_{\gamma \beta } .$ It signals a photonic replica-symmetric paramagnetic-like phase or a RSB glassy phase if its maximum occurs at *q*_max_ = 0 (no RSB) or at values |*q*_max_|≠ 0 (RSB), respectively^33^.

On the other hand, by considering in Eq. (1) the wavelength indexes $M = i$ and $N = j$ and the spectrum index $K = \gamma$, we write the Pearson correlation coefficient^44,45^ between intensity fluctuations at wavelengths $\lambda_{i}$ and $\lambda_{j} ,$

3 $$ P_{M = i,N = j} = C_{ij} = \frac{{\sum\nolimits_{\gamma } {\Delta_{\gamma i} \Delta_{\gamma j} } }}{{\sqrt {\left[ {\sum\nolimits_{\gamma } {\left( {\Delta_{\gamma i} } \right)^{2} } } \right]\left[ {\sum\nolimits_{\gamma } {\left( {\Delta_{\gamma j} } \right)^{2} } } \right]} }} . $$

We note that, differently from Eq. (2) in which the summations are over the wavelengths, in the Pearson coefficient $C_{ij}$ the sums are over the spectra $\gamma$ emitted at distinct times. In other words, Eq. (3) takes into account the dynamics evolution of the correlation between fluctuations of intensity at wavelengths $\lambda_{i}$ and $\lambda_{j} .$ Consider, for instance, that $\lambda_{i}$ and $\lambda_{j}$ represent the wavelengths of two modes of the laser system. Then a null value of $C_{ij}$ implies that these modes behave statistically in an uncorrelated way over the time interval of the spectra series. Conversely, a positive (negative) $C_{ij}$ signals that the statistical fluctuations in the intensity of one mode are positively (negatively) correlated to those in the other mode, so that the spatially overlapped coupled modes share (compete for) gain along the measurement.

**Figure 2:**
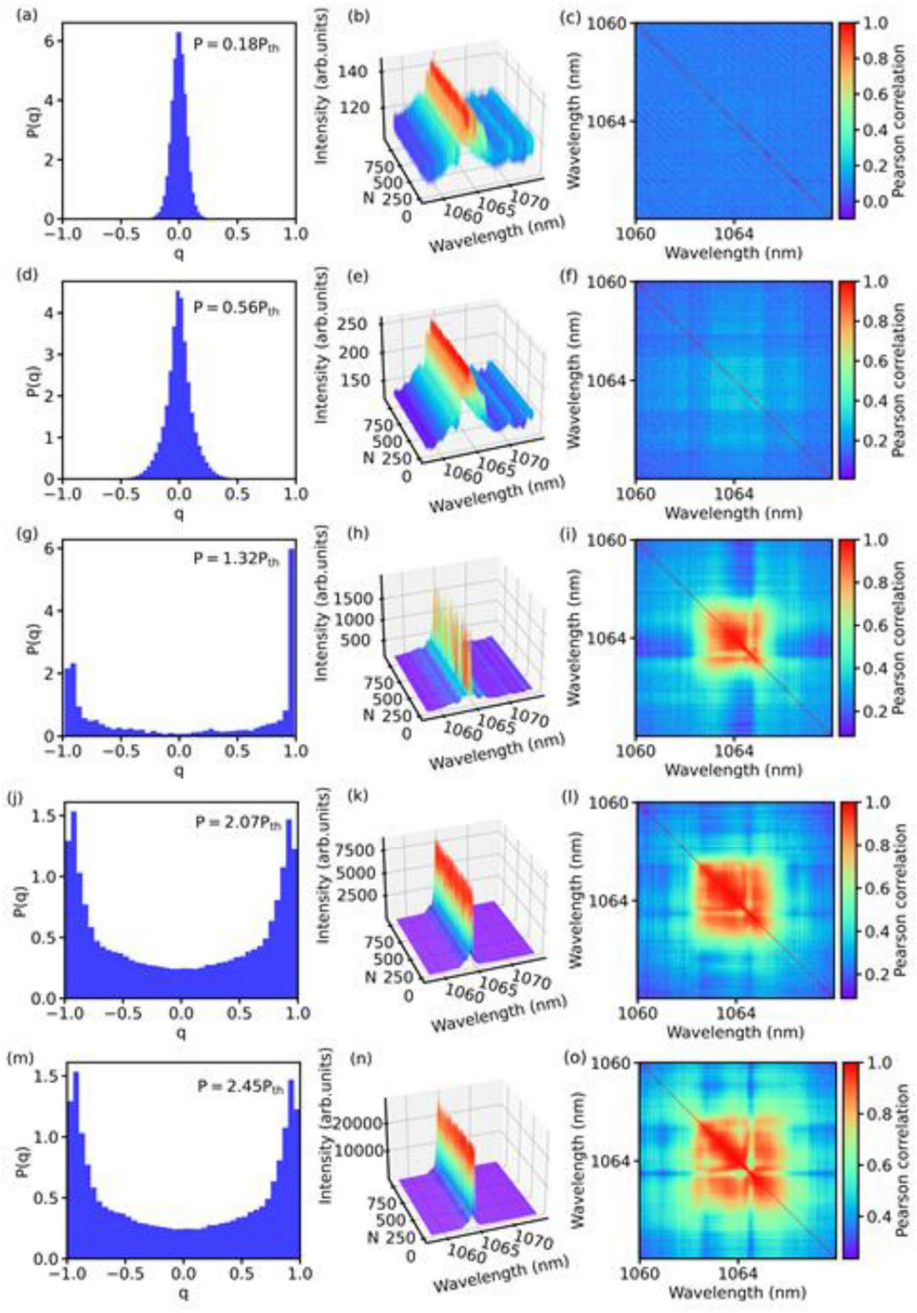
RSB behavior and Pearson correlations in the Nd:YAG RL. Distribution $P\left( q \right)$ of Parisi overlap parameter values (left column), Eq. (2), 1000 spectra profiles (center), and heatmap plots of the Pearson correlation coefficient (right column), Eq. (3). Each horizontal line corresponds to an excitation power, from below threshold (two upper lines: $P/P_{th} = 0.18$ and $P/P_{th} = 0.56$), to around (central line: $P/P_{th} = 1.32$), and well above threshold (two lower lines: $P/P_{th} = 2.07$ and $P/P_{th} = 2.45$). Below threshold, a replica-symmetric paramagnetic-like phase identified by a unimodal central peaked $P\left( q \right)$ coincides with weakly fluctuating spectra and uncorrelated intensity fluctuations with mostly blue heatmaps of the Pearson coefficient. Above threshold, the RSB glassy behavior sets in with two side peaks in $P\left( q \right)$ along with stronger intensity fluctuations and increasingly correlated modes (orange and red regions in the heatmaps).

Figure 2 displays three sets of vertical panels. The ones on the left column show the PDFs $P\left( q \right)$ of values of the Parisi overlap parameter; in the center we depict the profiles of 1000 recorded spectra; and on the right column we present results for the Pearson correlation coefficient $C_{ij}$. In particular, in the latter all cross-correlations between intensity fluctuations at distinct wavelengths, as well as self-correlations (thin diagonal red lines), are shown in color scale in the form of heatmap plots.

Each horizontal line in Fig. 2 corresponds to a value of the excitation power, from the prelasing regime well below threshold, with $P/P_{th} = 0.18$ and $P/P_{th} = 0.56$ (two upper lines), to the RL phase around the threshold with $P/P_{th} = 1.32$ (center line), and the RL regime well above threshold, $P/P_{th} = 2.07$ and $P/P_{th} = 2.45$ (two lower lines).

The simultaneous measure of the Parisi and Pearson parameters in Fig. 2, together with the analysis of the spectra profiles, helps to shed light on the role of the modes correlations to the emergence of the RSB glassy phase in the Nd:YAG RL. We first note in Fig. 2b,e that the spectra below threshold present rather weak intensity fluctuations in association with mostly blue heatmap patterns in the Pearson coefficient (Fig. 2c,f), evidencing that the fluctuations are fairly uncorrelated in the prelasing state. Consistently, the unimodal PDFs $P\left( q \right)$ centered around $q = 0$, seen in Fig. 2a,d, indicate the presence of a photonic replica-symmetric paramagnetic-like regime.

As the excitation power is increased and the threshold is approached, we observe in the central line of Fig. 2 the concomitant onset of correlations (orange and red in the heatmap of the Pearson coefficient $C_{ij}$, Fig. 2i), along with a strong bandwidth narrowing in the spectra with intensity peak near $\lambda = 1064$ nm and much larger intensity fluctuations, Fig. 2h. A simultaneous emergence of the RSB phenomenon takes place, as indicated in Fig. 2g by the presence of the side peaks in the bimodal $P\left( q \right)$.

Finally, as the excitation power is enhanced further (last two lines of Fig. 2), intensity fluctuations nail down (Fig. 2k,n) in the RSB regime above threshold displaying a bimodal $P\left( q \right)$ (Fig. 2j,m), with even stronger correlations between modes (larger red regions in Fig. 2l,o) that share the gain in an increasingly homogenous way. This phase is termed^28^ a self-averaged gain regime, with Gaussian distribution of emitted intensities. It contrasts with the Lévy-like intensity statistics observed near the threshold and the first prelasing Gaussian regime below threshold^28,29^.

Since modes overlap spatially and stochastically compete for gain, we note in Fig. 2i,l,o that this competition can favor some subsets of modes, displaying stronger Pearson cross-correlation values, while undermining others with lower correlation (less intense heatmap regions).

We now turn to the joint analysis of intermittency effects along with modes correlations and RSB in the Nd:YAG RL system.

Turbulence and RSB spin glass phenomena are among the most elusive problems in physics^51^. A task even more challenging is to study their intertwined properties in the quite rare event when they coexist simultaneously in any physical system^51^. Photonic turbulence-like behavior has been recently reported^49^ in a random fiber laser. Here we also demonstrate this phenomenon in a RL, and further analyze the onset and fading out of intermittency effects with respect to the emergence and persistency of modes correlations and RSB behavior.

In fluid turbulence^51^, the relevant statistical quantities are the velocity increments between two points in the fluid, rather than the velocities themselves. In analogy, here we analyze the intensity increments, $\delta I_{\tau ,\gamma i} = I_{\gamma + \tau ,i} - I_{\gamma i}$, between spectra $\gamma$ and $\gamma + \tau$ separated in time by ${\Delta }t = \tau t_{0} ,$ with integers $\gamma$ and $\tau ,$ and $t_{0} =$ 100 ms as the time interval between two consecutive spectra emitted by the Nd:YAG RL. For a proper statistical analysis including long separation times $\tau \gg 1$, we had to take into account a 5 × larger number of spectra (5000). Moreover, we conveniently define a variable with null average and unit variance, $x_{\tau ,\gamma i} = \delta I_{\tau ,\gamma i} /{\text{var}} \left\{ {\delta I_{\tau ,\gamma i} } \right\}$, where ${\text{var}}\left\{ {\delta I_{\tau ,\gamma i} } \right\}$ is the variance of the long time series of intensity increments. We also choose in our analysis the wavelength $\lambda_{i}$ of maximum intensity (once $\lambda_{i}$ is set, we drop the wavelength index $i$ hereafter).

On the one hand, when nonlinearities are not relevant, as in the replica-symmetric regime with uncorrelated intensity fluctuations below threshold, the intensity increments are statistically independent and the PDF $P\left( {x_{\tau } } \right)$ of $x_{\tau \alpha }$ values is a Gaussian for any separation time $\tau$ (in units of $t_{0}$). On the other hand, as the excitation power is increased above threshold, dynamical optical nonlinearities give rise to turbulent-like emission in the intensity fluctuations of spectra close in time ($\tau \approx 1$), causing $P\left( {x_{\tau } } \right)$ to develop a heavy tail in the asymptotic large-$x_{\tau }$ regime^48^. However, for large separation time scales $\tau \gg 1$ a Gaussian $P\left( {x_{\tau } } \right)$ still holds even above threshold, in similarity to fluid turbulence^51^, in which a heavy-tailed PDF of velocity increments sets in between nearby points, but a Gaussian PDF always arises when uncorrelated distant points are considered.

From the theoretical perspective, a hierarchical stochastic model was proposed in^49^ to explain the photonic turbulent-like behavior observed in an erbium-based random fiber laser. It was noticed that the PDF of intensity increments between consecutive spectra ($\tau = 1$) in the turbulent-like regime above threshold remains Gaussian but only for short time intervals in the long series of intensities, with a variance that fluctuates slowly in time. In this regime with $\tau = 1$, a heavy-tailed PDF $P\left( {x_{\tau } } \right)$ arises by integrating out these Gaussians along with the associated distribution of variances. The hierarchical model generally considered multiple time scales of variance fluctuations, in a way similar to Kolmogorov’s hypothesis of energy cascades and intermittency in fluid turbulence^51^. The intermittency of the stochastic flux of energy between the relevant time scales is a key ingredient to set the statistical properties of $P\left( {x_{\tau } } \right)$. Indeed, when the intermittent behavior is mitigated, for example, by decreasing the input excitation power and optical nonlinearity degree, the heavy tail of $P\left( {x_{\tau } } \right)$ fades away and a Gaussian PDF with a single scale variance emerges for any $\tau$ in the non-turbulent regime below threshold.

**Figure 3:**
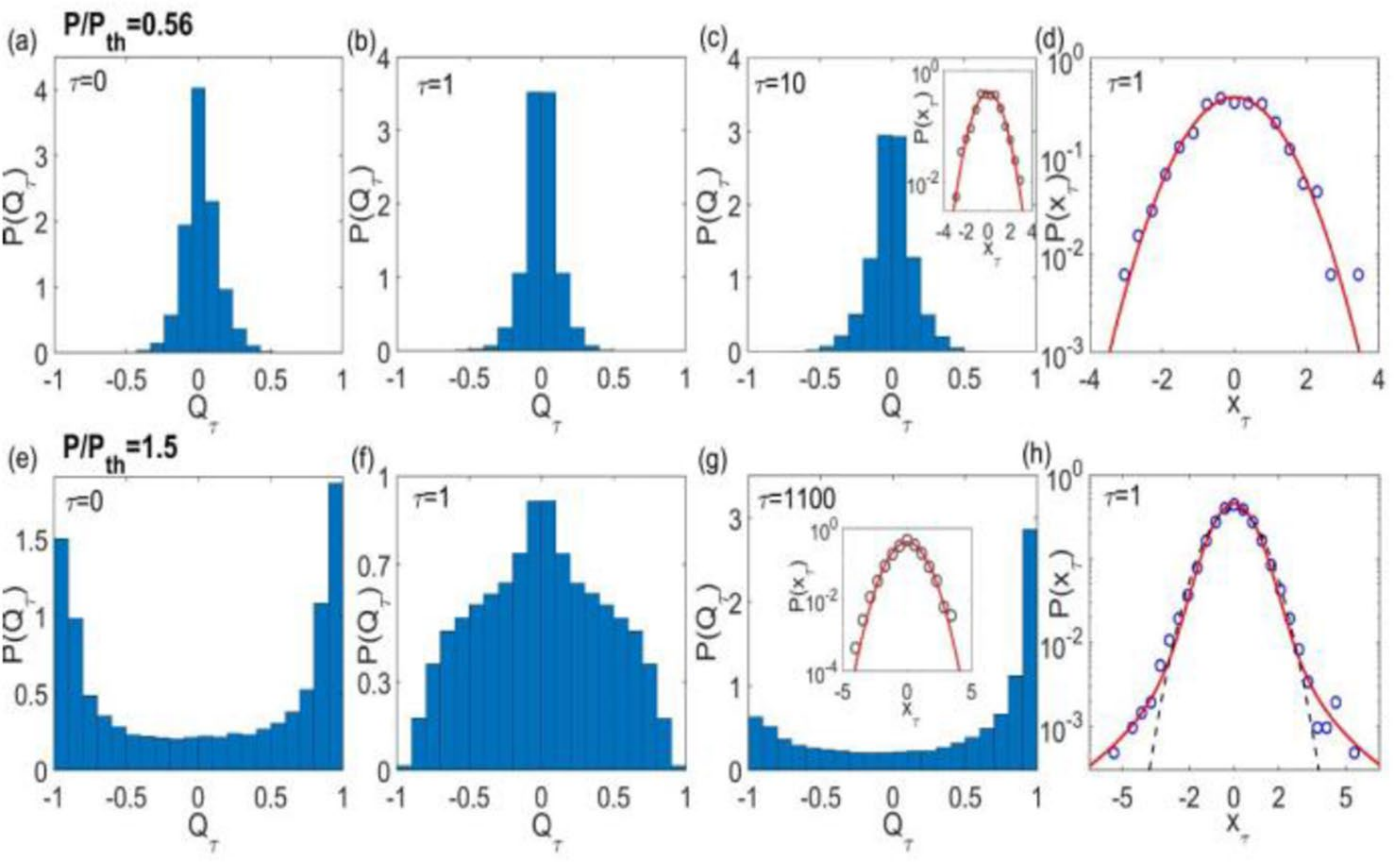
Intermittency effects and RSB behavior in the Nd:YAG RL. Distributions $P\left( {Q_{\tau } } \right)$ and $P\left( {x_{\tau } } \right)$ of the modified Pearson correlation coefficient, Eq. (4), and normalized intensity increments, $x_{\tau ,\gamma i} = \delta I_{\tau ,\gamma i} /{\text{var}}\left\{ {\delta I_{\tau ,\gamma i} } \right\}$, of spectra separated in time by $\tau t_{0} ,$ with $t_{0} = 100$ ms. The upper (lower) horizontal line corresponds to the excitation power $P/P_{th} = 0.56$ ($P/P_{th} = 1.50$) below (above) threshold. For $\tau = 0$ the PDF $P\left( {Q_{\tau } } \right)$ is equivalent to the Parisi overlap distribution $P\left( q \right)$, with (**a**) a single central peak in the replica-symmetric regime below threshold [also seen in (**b**)] and (**e**) double side peaks with RSB above threshold. For $\tau = 1$, in the latter (former) a Lévy (Gaussian) $P\left( {x_{\tau } } \right)$ indicates in (**h**), (**d**) the coexistence with a photonic turbulent-like (non-turbulent) regime above (below) threshold. For $\tau \gg 1$ only Gaussians are found [insets of (**c**) and (**g**)]. As the intermittency effects fade away in the crossover from the $\tau = 1$ to $\tau \gg 1$ time scales, a quite distinct statistical behavior of $P(Q_{\tau } )$ emerges, with the most likely values of $Q_{\tau }$ in (**f**) becoming the least probable ones in (**g**), and vice-versa.

Figure 3 shows in the right column and insets the PDF $P\left( {x_{\tau } } \right)$ of intensity increments in full agreement with the above discussion. Indeed, we notice a Gaussian $P\left( {x_{\tau } } \right)$ for $\tau \gg 1$ both below and above the threshold (insets of Fig. 3c,g), as well as for $\tau = 1$ below threshold (Fig. 3d), consistently with a non-turbulent behavior. On the other hand, for $\tau = 1$ above threshold (Fig. 3h) the PDF $P\left( {x_{\tau } } \right)$ is well fitted by a Lévy $\alpha$-stable distribution^24^, with asymptotic heavy-tail power-law decay $P\left( {x_{\tau } } \right) \sim 1/x_{\tau }^{\alpha + 1}$ and stability index $\alpha = 1.83$ (red solid line). For comparison, we show in black dashed line in Fig. 3h the unsuccessful fit attempt by a rapidly decaying $\alpha = 2$ Gaussian PDF, $P\left( {x_{\tau } } \right) \propto \exp \left( { - x_{\tau }^{2} /2} \right)$. These results sign for a photonic turbulent-like behavior of intensity emissions in the Nd:YAG RL above threshold.

The intermittency effects and RSB phenomenon can be analyzed jointly by defining another modified Pearson coefficient^50^ which is simultaneously sensitive to both photonic glassy and turbulent-like behaviors,

4 $$ Q_{\tau ,\gamma \beta } = \frac{{\sum\nolimits_{i} {d_{\tau ,\gamma i} d_{\tau ,\beta i} } }}{{\sqrt {\left[ {\sum\nolimits_{i} {\left( {d_{\tau ,\gamma i} } \right)^{2} } } \right]\left[ {\sum\nolimits_{i} {\left( {d_{\tau ,\beta i} } \right)^{2} } } \right]} }} . $$

In Eq. (4) the new fluctuation variables are defined as $d_{\tau ,\gamma i} = y_{\tau ,\gamma i} - y_{\tau i}$ and $y_{\tau ,\gamma i} = I_{\gamma + \tau ,i} - cI_{\gamma i}$, with ${\text{c}} = 0$ if $\tau = 0$ and ${\text{c}} = 1$ if $\tau > 0.$ We note that for $\tau = 0$ the fluctuations $d_{\tau ,\gamma i}$ coincide with those of the Parisi overlap parameter, ${\Delta }_{\gamma i}$ in Eq. (2), used to infer the RSB phenomenon. On the other hand, for $\tau > 0$ $d_{\tau ,\gamma i}$ is equivalent to the intensity increments $\delta I_{\tau ,\gamma i}$ in the analysis of photonic turbulence.

The first column in Fig. 3 displays the PDF $P(Q_{\tau } )$ for $\tau = 0$, indicating, just like in the first column of Fig. 2, a replica-symmetric (RSB) phase below (above) threshold with uncorrelated (correlated) intensity fluctuations, see Fig. 3a,e. Interestingly, the effect of intermittency can be also inferred from the plots in Fig. 3 by comparing the behavior of $P(Q_{\tau } )$ in the RSB regime for separation times $\tau = 1$ and $\tau \gg 1$ as follows.

We first note in Fig. 3g above threshold that the PDF $P(Q_{\tau } )$ for $\tau = 1100$ shows a bimodal profile similar to that of the Parisi overlap distribution $P\left( q \right)$ in the RSB regime. In fact, since the Parisi overlap parameter in Eq. (2) considers *all* separation times $\tau$ between spectra, we thus expect that the statistical weight of the replica overlaps with $\tau \gg 1$ dominates over the long time series of spectra. Thus, $P(Q_{\tau } )$ should actually appear qualitatively similar to the bimodal PDF $P\left( q \right)$ when the turbulence-like intermittency effects are suppressed for large separation time scales $\tau \gg 1$.

A remarkably distinct picture emerges for short separation times, as in Fig. 3f for $\tau = 1$. In this case, intermittency turbulent-like effects occur above threshold and a quite distinct statistical behavior of $P(Q_{\tau } )$ arises for $\tau = 1$, in contrast with the results for $\tau = 1100$ in Fig. 3g. For short time scales, the intermittency tends to increase the probability of events that are rarer at much larger scales. In other words, the most likely values of $Q_{\tau }$ when strong intermittency effects are present essentially become the least probable ones when intermittency fades away, and vice-versa. As a consequence, differently from the results for $\tau = 1100$ in Fig. 3g, the PDF $P(Q_{\tau } )$ displays in Fig. 3f a unimodal profile when $\tau = 1$, in agreement with the above discussion.

In summary, the above discussion can be separated into the analysis of the related photonic behaviors of the Nd:YAG RL below and above threshold. In the latter both RSB and turbulent-like phenomena coexist. The first is indicated by the double-peaked Parisi overlap parameter (Fig. 3e), while the second is signaled, e.g., by the long-tail behavior of $P(x_{\tau } )$ for $\tau = 1$ (Fig. 3h). As for the generalized Pearson coefficient, this implies a broad maximum in $P(Q_{\tau } )$ near $Q_{\tau } = 0$ for $\tau = 1$ but also with non-negligible values for $Q_{\tau } \ne 0$ (Fig. 3f). On the other hand, below threshold RSB cannot sustain, as seen from the single central maximum of the Parisi parameter in Fig. 3a. Correspondingly, turbulent-like behavior is absent, as shown in the Gaussian profile of $P(x_{\tau } )$ for $\tau = 1$ (Fig. 3d). In this case, $P(Q_{\tau } )$ for $\tau = 1$ shows a quite pronounced maximum at $Q_{\tau } = 0$ (Fig. 3b).

Although the theoretical approach^38^ to the RSB glassy phase in RLs assumes the thermodynamical limit in which the number *N* of modes tends to infinity, actual photonic systems in fact present values of *N* much away from this limit. For example, N ≈ 200 for our system and an erbium-based random fiber laser^34^ and N ≈ 300 for a random Raman laser^52^ and a RL based on a Rhodamine 6G dye solution with TiO_2_ particles^34^, although it has been also estimated that *N* ~ 10^10^ modes/mm^3^ in a liquid-state dye RL^53^. The use of finite-size-scaling techniques standardly applied to magnetic spin glasses^37^ thus emerges as an interesting perspective also for photonic systems with RSB glassy behavior. To our knowledge, no critical exponents associated with the glassy transition in these systems have been calculated so far and an ideal photonic glassy system for this purpose should be one in which the number of modes could be experimentally controlled.

In conclusion, among several striking features, RLs have been recently established as remarkable experimental platforms to study quite intricate phenomena in the multidisciplinary field of complex systems. Indeed, some of the most challenging current problems in physics find their counterparts and have been investigated in the context of RLs. Examples include photonic turbulence-like properties, spin-glass phase with RSB, and dynamics correlations between lasing modes. In the same pace, a number of statistical tools have been employed in these investigations, such as Lévy distributions^27–29^, Parisi overlap parameter^24,33^, and Pearson correlation coefficient^44–48^.

Our joint analysis of these complex photonic phenomena through a couple of modified Pearson correlation coefficients in the Nd:YAG RL showed how intertwined these behaviors are in a RL system, and indicate that they might share common physical underlying mechanisms. Although it is out of the scope of the present work to propose a theorical model to account for such phenomena, we suggest that a unique combination of key ingredients is subjacent to the mechanisms responsible for such behaviors, as the structural disorder in the distribution of gain and scattering elements, spatial overlap of multiple coupled modes, and suitable degree of optical nonlinearity, all of them present in the Nd:YAG RL analyzed here from a single set of measurements of intensity fluctuations. We hope our findings can stimulate the quest for the joint understanding of these complex behaviors under a unified theoretical approach, which can benefit from tools and concepts employed in the separate treatment of such challenging phenomena.

## Methods

### Sample preparation

To synthesize the particles of Nd:YAG (4.0% mol/mol to Y^3+^), we used^54,55^ 50 mL of 0.2 M boehmite sol co-doped with Nd^3+^ and Y^3+^ as a precursor and pyrolyzed in a Spray Pyrolysis (SP) system.

For the boehmite sol preparation, 12 ml of aqueous yttrium nitrate solution (0.5 M) and 4 ml of neodymium nitrate solution (0.1 M) were added to 20 ml of ultrapure water previously heated to 83 °C. Then, 2.43 g (0.01 mol) of the aluminum tri-sec-butoxide was added and hydrolyzed for 1 h under stirring, and 0.5 ml of nitric acid was added as a peptizing agent. The final volume was adjusted to 50 mL with ultrapure water after cooling the suspension. This 0.2 M sol was spray pyrolyzed into the SP system. In this setup, the aerosol was generated at an ultrasound chamber, where a 2.4 MHz frequency piezoelectric pellet was employed. Subsequently, the aerosol was transported by a gas flow (0.1 m^3^/h atmospheric air) through two heat treatment zones: the drying zone (150 °C) and the pyrolysis one (700 °C). In the first the solvent was evaporated and the initial solid particles were attained; and in the second the final material was obtained by a fast heat treatment. At the last step, the powder was collected in an electrostatic filter (150 °C) operating at 11 kV. Finally, the collected powder was further heated at 1100 °C for 12 h.

### Micron size particles and optical characterization

The Nd:YAG particles (4.0%) were characterized structurally and morphologically by X-ray diffractogram (XRD), FTIR and SEM, and the luminescent properties were studied by photoluminescence spectroscopy. The XRD showed peaks related to the structural planes of YAG, thus confirming that the YAG phase was obtained^56^.

**Figure 4:**
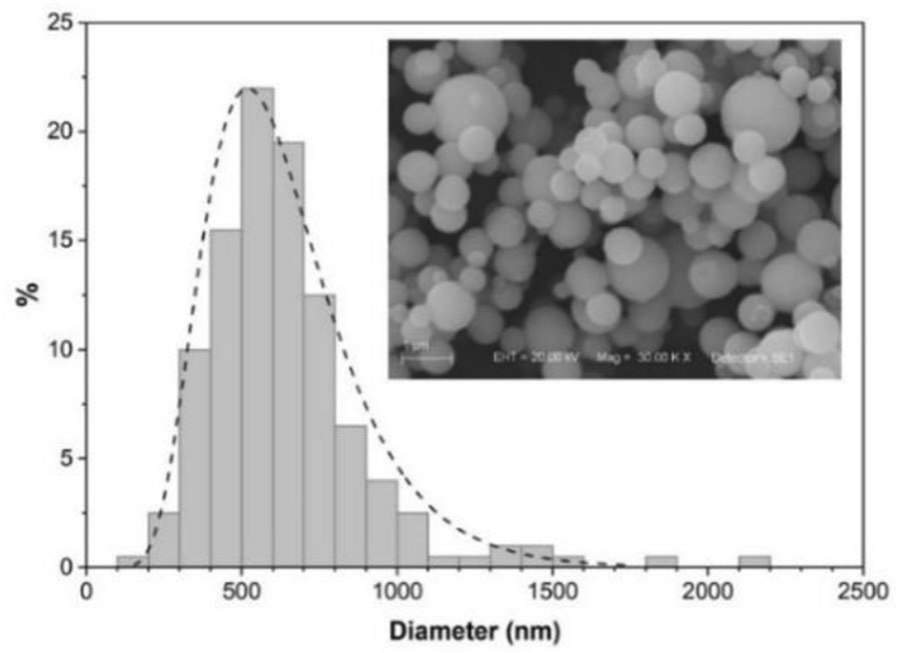
Characterization of Nd:YAG particles. Diameter distribution of micron size Nd:YAG (4.0%) particles synthesized by spray pyrolysis, with average of 0.6 μm. (Inset) Scanning electron microscopy image of the sample.

Figure 4 shows the typical morphology and particles size distribution of the powder sample used in this work. The sizes of the spherical particles ranged from 0.1 μm to 2.0 μm, with average diameter of 0.6 μm.

**Figure 5:**
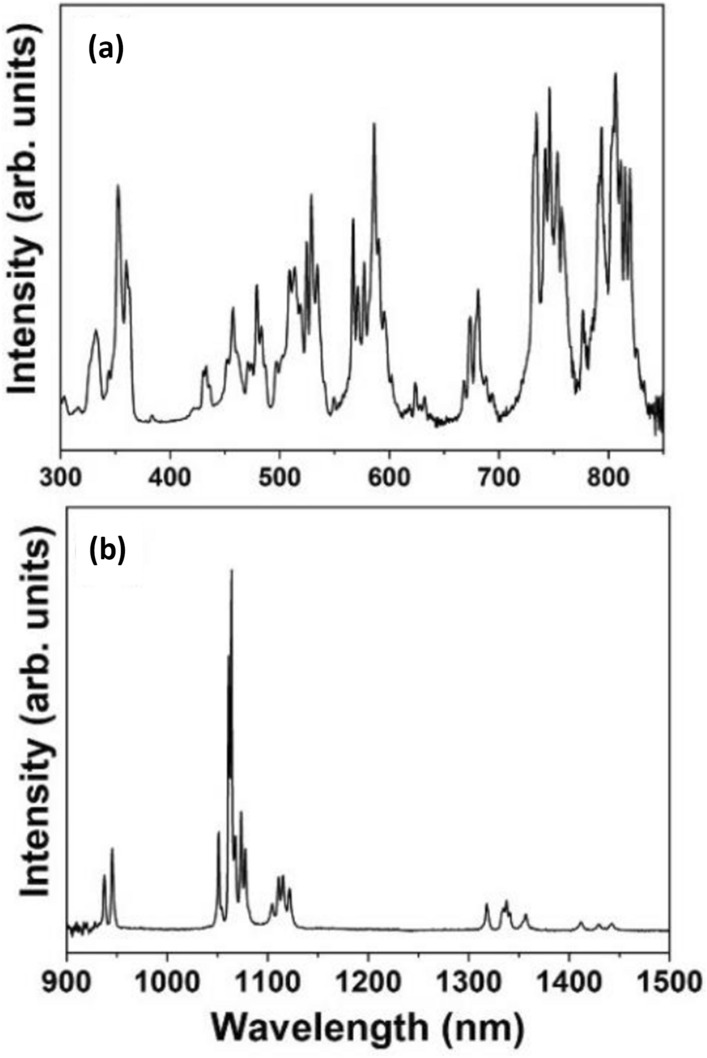
Photoluminescence characterization. (**a**) Excitation spectrum (emission band monitored at 1064 nm) and (**b**) emission spectrum (under excitation at 808 nm) of the Nd:YAG (4.0%) powder. The most important emission 1064 nm corresponds to the Nd^3+^ transition ^4^F_3/2_ → ^4^I_11/2_.

The photoluminescence analyses were performed on a Fluorolog 3 Horiba Scientific (model FL3-22) equipment with dual excitation and emission monochromators and Hamamatsu R928 (visible) and H10330-75 (near infrared) photomultipliers, with results shown in Fig. 5. The photoluminescence spectrum obtained under continuous-wave excitation at 808 nm exhibits narrow and well-resolved bands characteristic of the Nd^3+^ f-f transitions in the YAG crystalline structure, in which the Nd^3+^ ions replace the Y^3+^ ones in dodecahedral sites with D_2_ symmetry^57^.

### Random laser setup and characterization

**Figure 6:**
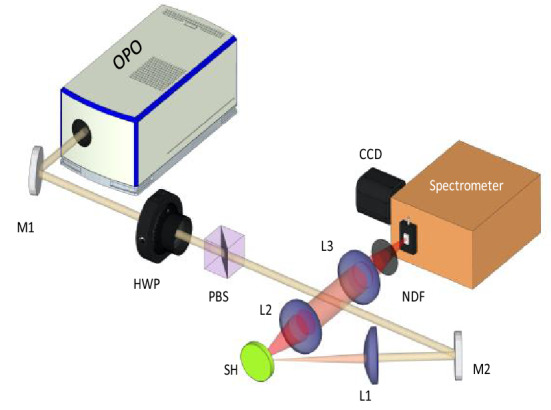
Experimental setup for measurements in the Nd:YAG RL. OPO is an optical parametric oscillator, M1 and M2 are 100% reflecting mirrors @800 nm, HWP is a half-wave plate @800 nm, and PBS is a broadband polarizer beam splitter. Together they control the pump energy. L1, L2, L3 are converging lens. SH is the sample holder, NDF are neutral density filters, and the data is acquired by a high-resolution spectrometer with CCD (see text for equipment details).

The experimental setup shown in Fig. 6 is typically used for powder-based RL studies. The pump source was an optical parametric oscillator (OPO, Opotek) pumped by the second harmonic of a Q-switched Nd:YAG laser and tuned to the 810 nm emission wavelength. A repetition rate of 10 Hz was employed and the source pulse duration was 5 ns.

We used a high-resolution spectrometer (SpectraPro 500, Acton Research Corporation) coupled with a charge-coupled device (CCD), covering, in real time, the range from 1040.024 nm to 1070.836 nm. In the discretization of the spectra the bin width was 0.032 nm, a value that corresponds to the resolution of the acquisition system.

To study the RSB behavior and modes correlations, we acquired a series of 1000 spectra for each input excitation energy, and for intermittency effects 5000 spectra were obtained in the regimes below, close to, and well above the threshold.

## Data Availability

All relevant data are available from the authors.
